# Multi-omics analysis reveals galactose metabolism as a key regulatory pathway of stress adaptation to magnesium deficiency in passion fruit

**DOI:** 10.3389/fpls.2026.1878176

**Published:** 2026-06-26

**Authors:** Wei Li, Qiufang Zhu, Xiwen Hao, Biao Huang, Jiuliang Xu, Tong Wang, Ziwen Ren, Xuexian Li, Liangquan Wu, Fusuo Zhang

**Affiliations:** 1 Fujian Academy of Agricultural Sciences, Institute of Quality Standards & Testing Technology for Agro-Produets, Fujian Key Laboratory of Agro-Products Quality & Safety, Fuzhou, Fujian, China; 2 International Magnesium Institute, College of Resources and Environmental Sciences, Fujian Agriculture and Forestry University, Fuzhou, Fujian, China; 3 College of Resources and Environmental Sciences, China Agricultural University, Beijing, China

**Keywords:** fruit quality, galactose metabolism, magnesium deficiency, metabolomics, *Passiflora edulis*, transcriptomics

## Abstract

Magnesium (Mg) deficiency is a major nutritional constraint in acidic orchard soils and can impair both vegetative growth and fruit quality in passion fruits (*Passiflora edulis*). However, the physiological and molecular mechanisms underlying Mg deficiency responses in passion fruit remain insufficiently understood. This study employed a comprehensive approach, integrating physiological assessments with metabolomic and transcriptomic profiling, to explore the responses to Mg deficiency in the leaves and fruits of purple passion fruit. Our findings revealed that Mg deficiency detrimentally affected chloroplast ultrastructure, significantly decreased SPAD values by an average of 16.1% and net photosynthetic rates by an average of 55.0% in leaves, and led to the accumulation of starch and soluble sugars (increased by approximately 26.4% and 11.9%, respectively) in source leaves. In fruit peels, Mg deficiency resulted in a significant reduction in anthocyanin content by approximately 46.3% and a 2.4-fold increase in chlorophyll content, leading to a 79.5% decrease in the anthocyanin-to-chlorophyll ratio and producing a commercially undesirable green coloration. Through integrative multi-omics analysis, we identified the specific alteration of the galactose metabolism pathway under Mg deficiency, which facilitated the synthesis of melibiose, fructose, and succinate as potential osmoprotectants. These results suggest that Mg deficiency compromises both the marketable appearance and nutritional quality of passion fruit, while simultaneously triggering carbon reallocation as an underlying potential mechanism. In conclusion, the metabolism of galactose may enhance plant adaptation to magnesium deficiency stress, thereby offering a physiological foundation for developing optimal magnesium fertilization strategies in passion fruit orchards cultivated in acidic soils.

## 1 Introduction

Passion fruit *(Passiflora edulis)* is a fast-growing vine that can fruit within the first year after planting, making it an ideal model for fruit quality studies (Chen et al., 2025c; Sun et al., 2025). It is rich in bioactive compounds, such as vitamin C, flavonoids, and terpenoids (Chen et al., 2025c; Li et al., 2025; Luo et al., 2025), which contribute to its high nutritional and commercial value (Muller et al., 2015; Silva and Bottoli, 2015; Corrêa et al., 2016; Guimarães et al., 2024). Currently, Brazil is the largest producer and consumer of passion fruit in the world (Da Silva Santos et al., 2024; Chen et al., 2025c). With the increasing demand for passion fruit, both planting area and yield have expanded substantially in China. Magnesium (Mg) is critical for plant growth and quality. However, exchangeable Mg content in soils is commonly low in major cultivation areas, including Brazil and China (Moreira and Fageria, 2009; Ishfaq et al., 2022). Therefore, understanding the effects of Mg on the growth and quality of passion fruit is of great importance.

Mg is a pivotal element for fruit growth and quality, as it is involved in chlorophyll biosynthesis and the activation of numerous enzymes (Ishfaq et al., 2022; Ali et al., 2024). These enzymes are involved in multiple metabolic pathways, including fatty acid, carbon, and nitrogen metabolism (Tian et al., 2023; Han et al., 2024). Mg can increase fruit yield. Previous studies have demonstrated that Mg application enhances the fruit yield of navel orange (Liu et al., 2022), ‘Jonagold’ apple (Bennewitz et al., 2011), and banana (Li et al., 2023). Moreover, Mg promotes carbohydrate accumulation in navel orange (Liu et al., 2022), loquat (Ali et al., 2024), and ‘*Feizixiao*’ Litchi (Peng et al., 2022). In addition, Mg promote color changes in citrus peel (Liu et al., 2022; Liu et al., 2023a), and regulates the accumulation of carotenoids (Liu et al., 2023b) and flavonoids (Xiong et al., 2023) in citrus fruits. Collectively, these findings indicate that Mg plays a crucial role in shaping both the external appearance and internal quality of fruits.

Mg has been reported to affect photosynthesis, quality, and yield in passion fruit. Mg deficiency affected the photosynthesis rates of purple passion fruit (Cárdenas-Pira et al., 2021). In banana passion fruit (*Passiffora tripartita* var. *mollissima*), magnesium stress affects dry weight accumulation and allocation, decreases leaf numbers leaf area (Lizarazo et al., 2013). The concentration of calcium and Mg in irrigation water exerted a major impact on passion fruit production variables, particularly at 259 days post-transplanting in yellow passion fruit (Francisco et al., 2021). Previous studies have found that Mg deficiency increases oxidative stress in plants, leading to changes in secondary metabolites in five passion fruit varieties (Hernandez-Martinez et al., 2023). Despite extensive research on the influence of Mg on fruit yield, sugar content, and acidity, there has been comparatively limited focus on its regulation of secondary metabolites, which are vital for both human health and the sensory quality of fruit. Moreover, the metabolic and molecular mechanisms underlying Mg-mediated regulation of fruit quality remain inadequately understood. Passion fruit (*Passiflora edulis’Tainong’*) is acknowledged as both a medicinal and edible fruit, rich in bioactive secondary metabolites. However, the specific metabolites affected by Mg and the mechanisms by which Mg regulates their biosynthesis are largely unknown. Traditional physiological methods are inadequate for capturing the intricate regulatory networks involved. Consequently, this study utilized an integrated transcriptomic and metabolomic approach to systematically analyze the molecular and metabolic responses of purple passion fruit to Mg deficiency. By correlating gene expression changes with metabolite accumulation, we aim to identify key pathways and regulatory hubs through which Mg affects fruit quality. The findings will provide a theoretical foundation for the strategic application of Mg fertilizer to enhance the quality of passion fruit.

## 2 Materials and methods

### 2.1 Plant material and treatments

Uniform seedlings of *Passiflora edulis ‘Tainong’* at the 7–8 leaf stage were transplanted into 250 L pots (three seedlings per pot) filled with a mixture of washed quartz sands and perlite (1:2, v/v). Prior to treatment, seedlings were precultured with a modified Hoagland nutrient solution. Seedlings were cultivated in a greenhouse under 14 h light/10 h dark photoperiod, with a relative humidity of 50-70% and day/night temperature of 35/20 °C. When new leaves appeared, the plants were washed several times with deionized water. Based on our preliminary experiments and previous studies in purple passion fruit by Cárdenas−Pira et al (Cárdenas-Pira et al., 2021), plants were supplied with nutrient solution containing either 0.01 mmol/L MgSO_4_ (Mg deficiency, −Mg) or 1 mmol/L MgSO_4_ (control, CK), as the 0.01 mmol/L concentration induces typical Mg deficiency symptoms (e.g., interveinal chlorosis) without causing plant death or severe growth arrest. Nutrient solutions were renewed every two days before flowering and every five days during the later growth stage. Photosynthetic parameters were measured after interveinal chlorosis appeared on leaves (same position N15, N20, and N25) under Mg-deficiency. These leaves were collected and fixed in glutaraldehyde and subsequently examined by electron microscopy. The pollination time of the fruits was recorded, and fruits entering the color-turning stage were bagged using non-shading mesh bags. Fruits were harvested after natural abscission at full maturity. Each treatment consisted of 18 seedlings (six pots) arranged in a completely randomized design. Physiological measurements, including SPAD, net photosynthetic rate, and the contents of soluble sugars and starch, were conducted using three biological replicates per treatment. For metabolomic analysis, six biological replicates were utilized per treatment, with each replicate consisting of fruit pulp collected from three seedlings grown in the same pot. In the case of transcriptomic analysis, the six metabolomic samples were randomly paired to create three pooled replicates, with each pool combining two biological replicates. The same biological samples were employed for both metabolomic and transcriptomic assays to ensure direct comparability of multi-omics data.

### 2.2 Phenotypic traits and physiological parameter determination

The chlorophyll relative content (SPAD value) was measured on the 20th and 25th leaves counting from the base of the plant using a SPAD-502 Plus chlorophyll meter (Konica Minolta, Japan). For each leaf, measurements were taken at the tip, middle, and basal parts, and the average value was recorded as the SPAD value of that leaf. Net photosynthetic rate was determined using an LI-6400 portable photosynthesis system (LI-6400; LI-COR, Lincoln, NE, USA). To determine the soluble sugar and starch contents, 0.1 g of freeze-dried leaf powder was subjected to extraction using 80% ethanol at 80 °C for 30 minutes, a process which was repeated three times. The resulting supernatant was combined for the quantification of soluble sugars via the anthrone-sulfuric acid colorimetric method. The remaining residue was utilized for starch analysis, wherein starch was gelatinized in distilled water at 100 °C for 15 minutes, subsequently digested with 9.2 M perchloric acid, and quantified as glucose equivalents using the same anthrone method. The contents of soluble sugar and starch were expressed in mg·g^-^¹ dry weight (DW). The anthocyanin content in passion fruit peel was quantified employing the pH-differential method. Peel samples, weighing 0.5 g, were extracted with 10 mL of 1% hydrochloric acid in methanol at a temperature of 4 °C for a duration of 24 hours under dark conditions. Following centrifugation at 10,000 g for 10 minutes at 4 °C, the absorbance of the supernatant was recorded at wavelengths of 530 nm and 700 nm in buffer solutions of pH 1.0 (0.025 M KCl) and pH 4.5 (0.4 M sodium acetate). The total anthocyanin content was expressed as cyanidin-3-O-glucoside equivalents (mg·g^-1^ dry weight), utilizing a molar extinction coefficient of 26,900 L·mol^-1^·cm^-1^ and a molecular weight of 449.2 g·mol^-1^. The chlorophyll content in the peel was quantified utilizing the alcohol-acetone extraction technique. Specifically, peel samples weighing 0.2 grams were homogenized in 5 milliliters of 80% acetone (v/v) and subsequently centrifuged at 5,000 g for 5 minutes. Absorbance measurements were conducted at wavelengths of 663 nm and 645 nm. The concentrations of chlorophyll a and b (μg·mL^-^¹) were determined using the equations established by Arnon (1949), and the total chlorophyll content was reported as mg·g^-^¹ fresh weight (FW).

### 2.3 SEM and TEM methods for observing cell structure

First, one leaf at the same position was collected from each plant grown under Mg deficiency treatment or Mg-sufficient treatment. Leaf pieces (0.5×0.5 cm) were excised from the middle of the leaf using a scalpel. Samples were prefixed with 2.5% glutaraldehyde and then stored at 4 °C, followed by postfixation in 1% osmic acid. Specimens were dehydrated through a graded ethanol series. For SEM (Scanning electron microscopy), part of the samples was subjected to critical-point drying in CO_2_ and coated with gold in a metallizer. Subsequently, starch grains in intracellular transverse sections of leaves were observed and photographed using a scanning electron microscopy (JSM-6380LV, Jeol Co, Tokyo, Japan). For TEM (Transmission electron microscope), the remaining samples were embedded in resin, sectioned using an ultrathin microtome, and double stained with 3% uranyl acetate-lead citrate. Finally, chloroplast ultrastructure was observed and photographed using a transmission electron microscope (HT7700, Hitachi, Tokyo, Japan).

### 2.4 Sample preparation and LC-MS/MS analysis

Untargeted metabolomic profiling was conducted on rumen fluid and seminal plasma samples utilizing a liquid chromatography-tandem mass spectrometry (LC-MS/MS) system. For the preparation of samples, 100 µL of each sample was combined with 500 µL of a methanol: acetonitrile (1:1, v/v) solution, followed by vortexing for 30 seconds and sonication for 10 minutes. The mixture was subsequently incubated at –20 °C for 1 hour and subjected to centrifugation at 10,000× g at 4 °C for 15 minutes. Thereafter, 500 µL of the supernatant was transferred to a new tube, to which 160 µL of acetonitrile: water (1:1, v/v) was added, and the mixture was vortexed for an additional 30 seconds. Following a second centrifugation step, 120 µL of the resultant supernatant was transferred into a 2 mL sample vial, and 10 µL of this solution was injected for analysis.

The liquid chromatography-tandem mass spectrometry (LC-MS/MS) system utilized a Waters Acquity I-Class PLUS ultra-high-performance liquid chromatography (UHPLC) apparatus, which was interfaced with a Waters Xevo G2-XS QToF high-resolution mass spectrometer. Chromatographic separation was achieved using a Waters Acquity UPLC HSS T3 column, characterized by a particle size of 1.8 µm and dimensions of 2.1 × 100 mm. The mobile phase A consisted of 0.1% formic acid in water, while mobile phase B comprised 0.1% formic acid in acetonitrile. The injection volume employed was 1 µL.

Mass spectrometry data were obtained using the MSe mode, managed by MassLynx V4.2 software, which facilitated the concurrent acquisition of both precursor and fragment ion information within a single analytical run. To ensure comprehensive compound detection, the initial mass spectrometry acquisition utilized a broad precursor isolation window. Data collection was conducted in dual-channel mode, employing a low collision energy of 2 V and a ramped high collision energy ranging from 10 V to 40 V. The scan rate was set at 0.2 seconds per spectrum. The electrospray ionization (ESI) source parameters were configured as follows: a capillary voltage of 2000 V in positive ion mode and -1500 V in negative ion mode; a cone voltage of 30 V; an ion source temperature of 150 °C; a desolvation gas temperature of 500 °C; a cone gas flow rate of 50 L/h; and a desolvation gas flow rate of 800 L/h.

The raw data were processed utilizing Progenesis QI software (version 4.0) to facilitate peak detection, alignment, and normalization. Metabolite identification was conducted through a systematic filtration process that integrated precursor ion accurate mass deviation, isotopic abundance, isotopic spacing, MS/MS matching score, and, when available, retention time alignment with authentic standards. The identification outcomes were cross-referenced with the integrated online METLIN database and a custom-developed in-house library. The final compilation of identified compounds exhibited a mass deviation of ≤10 ppm. Orthogonal partial least-squares discriminant analysis (OPLS-DA) and Kyoto Encyclopedia of Genes and Genomes (KEGG) pathway enrichment analyses were executed using the BMK Cloud platform (www.biocloud.net).

### 2.5 RNA extraction and RNA-Seq analysis

Total RNA was extracted from each sample using TRIzol Reagent (Life Technologies, California, USA) following the manufacturer’s instructions. RNA concentration and purity were assessed with a NanoDrop 2000 (Thermo Fisher Scientific, Waltham, USA), and RNA integrity was confirmed using an Agilent Bioanalyzer 2100 (Agilent Technologies, Santa Clara, CA, USA). For library construction, 1 μg of total RNA per sample was used. Poly(A) mRNA was isolated using NEBNext Poly(A) mRNA Magnetic Isolation Module (NEB, E7490), and then fragmented in the presence of divalent cations. First−strand cDNA was synthesized with random hexamer primers and reverse transcriptase, followed by second−strand cDNA synthesis using the NEBNext Ultra™ RNA Library Prep Kit for Illumina (NEB, E7530). The resulting double−stranded cDNA fragments were end−repaired, 3′−adenylated, and ligated to NEBNext adaptors (NEB, E7500). Size selection of approximately 240 bp fragments was performed using AMPure XP beads (Beckman Coulter, Inc.). After adaptor ligation, USER enzyme was applied, and the library fragments were PCR−amplified using high−fidelity DNA polymerase with universal and index primers. The final PCR products were purified with AMPure XP beads. Library quality and size distribution were verified on an Agilent Bioanalyzer 2100. Indexed libraries were pooled, clustered on a cBot system using the TruSeq PE Cluster Kit v4, and subjected to paired−end sequencing on an Illumina HiSeq platform to generate raw reads. Using the genome of *P. edulis*, a cultivar of purple passion fruit as the reference genome as the reference genome (https://ngdc.cncb.ac.cn/gwh/Assembly/17982/show).

### 2.6 Statistical and bioinformatic analysis

All statistical analyses were performed using R software (version 4.2.0) and the BMKCloud platform (https://www.biocloud.net/fxpt/app). Intra-group repeatability and quality control sample stability were evaluated using principal component analysis (PCA) and Spearman correlation analysis. Identified compounds were annotated by querying the KEGG, HMDB, and Lipidmaps databases.

For metabolomic data, orthogonal partial least squares discriminant analysis (OPLS−DA) was performed using the R package ‘ropls’. Model reliability was validated by 200 permutation tests (random re−assignment of group labels) to assess overfitting; the Q² intercept and the distribution of permuted R² and Q² values were examined. Variable importance in projection (VIP) scores were derived via seven−fold cross−validation. Differential metabolites between Mg−deficiency and control groups were screened using Student’s t−test (two−tailed). To control the false discovery rate (FDR) due to multiple comparisons (454 metabolites), the Benjamini–Hochberg procedure was applied. The combined criteria for selecting differentially accumulated metabolites (DAMs) were: |log_2_(FC)| ≥ 1, p < 0.05, and VIP > 1.

For transcriptomic data, differential expression analysis between the two groups was performed using the DESeq2 package (version 1.36.0). Genes with |log_2_(FC)| ≥ 1, p < 0.05 were considered differentially expressed genes (DEGs). All analysis such as Volcano plot, Venn, KEGG enrichment analysis (Kyoto Encyclopedia of Genes and Genomes), KOG (Eukaryotic Orthologous Groups), GO (Gene Ontology) and Network analysis were used by BMKCloud platform (https://www.biocloud.net/fxpt/app) for DAMs and DEGs.

All bioinformatic analyses were performed using the BMKCloud platform (https://www.biocloud.net/fxpt/app).

## 3 Results

### 3.1 Phenotypic and physiological responses of passion fruit leaves to Mg deficiency

**Figure 1:**
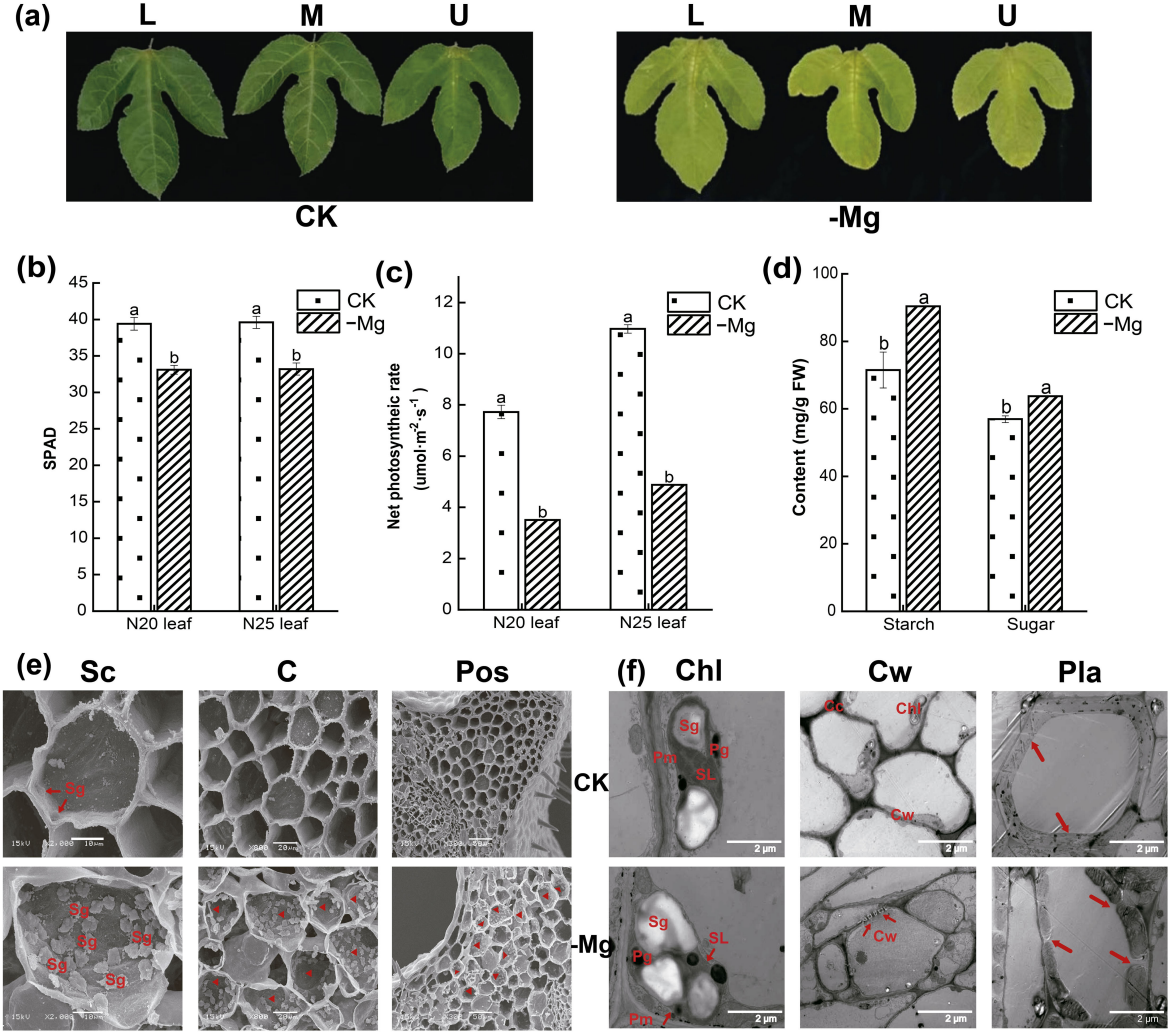
Phenotypic, ultrastructural and physiological responses of passion fruit leaves to Mg deficiency. **(a)** Interveinal chlorosis in middle and lower leaves (L, M, U). **(b)** SPAD value **(c)** net photosynthetic rate **(d)** starch and sugar contents in leaves **(e)** SEM images revealing single cell (Sc), starch-granule cell count (C), starch-containing regions (Pos) starch grain (Sg). **(f)** TEM images revealing chloroplast (Chl), cell wall (CW), plasmalemma (Pla), SL: stroma lamellae (SL), plastoglobule (Pg) plasma membrane (Pm), starch grain (Sg).

To validate the experimental treatments, Mg concentrations were quantified in the 20th and 25th leaves and Mg deficiency decreased them by approximately 25.5% and 39.0%, respectively, compared with the control (**Supplementary Figure 1A**). As anticipated, plants with adequate Mg levels demonstrated significantly elevated foliar Mg concentrations at both leaf positions in comparison to Mg-deficiency plants, thereby confirming the successful establishment of the intended experimental conditions. Mg deficiency induced leaf interveinal chlorosis (**Figure 1a**). Vein enlargement and corkiness were observed in the middle and lower leaves, whereas these symptoms were much less pronounced in the upper leaves. Mg deficiency significantly decreased SPAD values by an average of 16.1% and net photosynthetic rates by an average of 55.0% in leaves (**Figures 1b, c**). Compared with the control, Mg deficiency significantly increased leaf starch and total soluble sugar contents by approximately 26.4% and 11.9%, respectively (**Figure 1d**). As observed by SEM, there was a strong increase of starch grain (Sg) number and size in mesophyll cell of passion fruit under Mg deficiency (**Figure 1e**). Moreover, the cells with more starch grains clustered in the thick angular tissue near the veins (Pos). Mg deficiency significantly affected leaf ultrastructure (**Figure 1f**). Under Mg sufficient conditions, chloroplasts (Chl) displayed well-organized thylakoid membranes with clearly defined grana and stroma lamellae (SL), as well as intact double-envelope membrane systems. In contrast, Mg deficiency leaves exhibited disrupted chloroplast (Chl) membranes, poorly stacked thylakoids, and the presence of numerous plastoglobules (Pg). Additionally, varying degrees of damage to the cell wall (CW) and plasma membrane (Pm) were observed in leaf cells under Mg deficiency conditions. These results demonstrate that Mg deprivation leads to chlorosis and physiological response in passion fruit leaves, consistent with the typical character of Mg deficiency in the field.

### 3.2 Metabolic profiling of passion fruit under Mg deficiency stress

**Figure 2:**
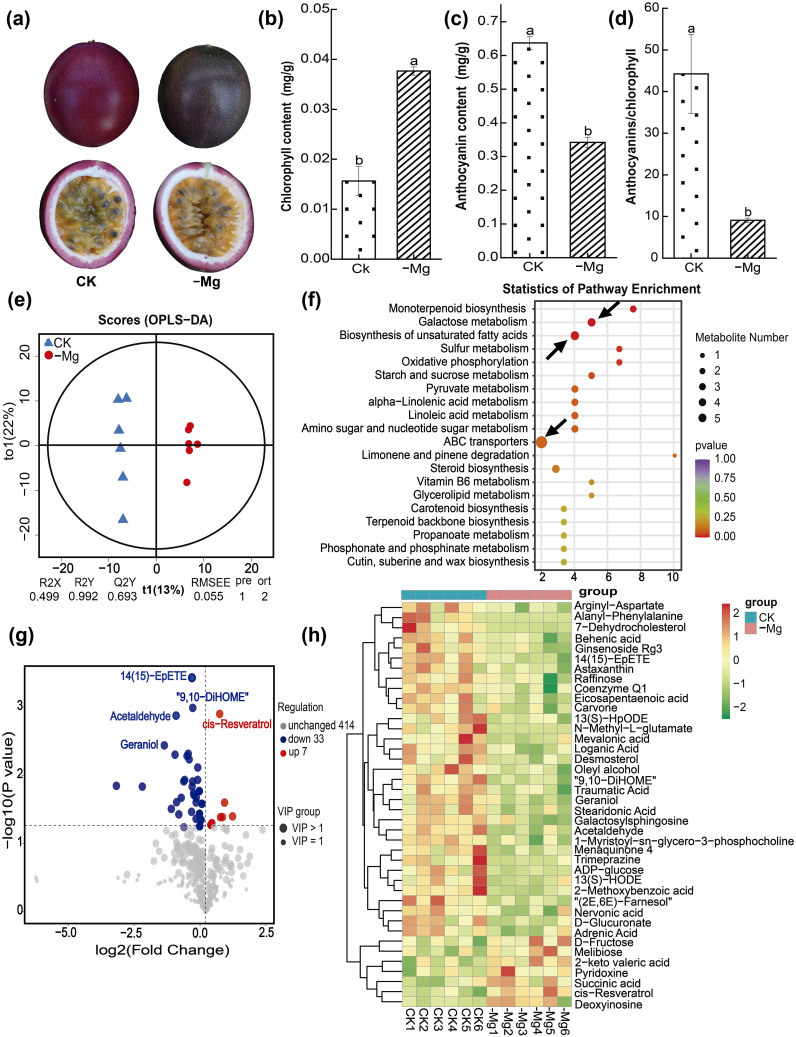
Pericarp color, physiological parameters and metabolomic analysis of passion fruit pulpunder Mg deficiency **(a)** surface and pulp phenotype **(b-d)** Chlorophyll and anthocyanin contents and their ratioin outer epidermis. **(e)** OPLS-DA score plot of pulp metabolites between Mg treatments. **(f)** Top 20 enriched KEGG pathways **(g)** Volcano plots of metabolites under different Mg levels. **(h)** Heat map of differential metabolites (CK vs. -Mg). CK: Mg treatment; -Mg: Mg deficiency.

Compared with the control, Mg concentration in passion fruits under Mg deficiency was significantly reduced by approximately 16.5%, confirming the effectiveness of the experimental nutrient design (**Supplementary Figure 1B**). The fruit rind colors were significantly different between different Mg treatments. Fruit of Mg deficiency treatment plants showed a dusky red rind, while the fruit of Mg treatment plants displayed bright red (**Figure 2a**). The effects of different treatments on anthocyanin and chlorophyll contents in the fruit peel are shown in **Figures 2b–d**. Compared with the control, Mg deficiency resulted in a significant reduction in anthocyanin content by approximately 46.3% in the peel. In contrast, chlorophyll content was significantly increased by about 2.4-fold under Mg deficiency. Consequently, the anthocyanin-to-chlorophyll ratio in the fruit peel was significantly reduced by approximately 79.5%compared with the Mg sufficient treatment.

The metabolite data of passion fruit pulp were analyzed according to the OPLS−DA model. The OPLS−DA scores plot showed excellent separation between Mg deficiency and normal treatment (**Figure 2e**). The OPLS-DA model parameters R_2_Y and Q_2_Y were 0.992 and 0.693, respectively, indicating that the model was stable and reliable. Using LC-MS, a total of 454 metabolites in passion fruit pulp were detected in positive and negative ion modes (**Supplementary Figure 3**). Then, 40 differential metabolites were selected based on the following parameters: VIP ≥ 1, |log_2_(fold change)| ≥ 1, and P-value ≤ 0.05. Only 7 differential metabolites were significantly up-regulated in Mg deficiency treatment, the rest of 33 differential metabolites were significantly down-regulated (**Figure 2g**). Many lipid classes were significantly down-regulated in Mg deficiency treatment, such as behenic acid, eicosapentaenoic acid, traumatic acid, stearidonic acid, 13(S)-HpODE, 13(S)-HODE. Under Mg stress treatments, amino acids, sugars, and terpenoids were significantly changed, including arginyl-aspartate, alanyl-phenylalanine, raffinose, D-glucuronate, loganic acid, geraniol, (2E,6E)-farnesol. Succinic acid of passion fruit pulp was significantly up-regulated in the Mg deficiency treatment. Under Mg stress, some metabolites contributing to aromas persistence were down-regulated, such as geraniol, acetaldehyde, and (2E,6E)-farnesol. Geraniol has a rose-like scent, and (2E,6E)-farnesol has a pleasant floral scent reminiscent of lily of the valley. These metabolic alterations imply that sweet and floral aromas might be diminished under conditions of magnesium deficiency; however, this hypothesis necessitates further validation through sensory evaluation experiments. Subsequently, metabolic pathway enrichment analysis was conducted on the differential metabolites between the two treatments. Bubble plots were generated for the top 20 enriched KEGG pathways (**Figure 2f**). The results showed that the significantly enriched pathways in the comparison between Mg deficiency and normal treatments included “Monoterpenoid biosynthesis (ko00902)”, “Galactose metabolism (ko00052)”, “Biosynthesis of unsaturated fatty acids (ko01040)”, “Oxidative phosphorylation (ko00190)”, “Sulfur metabolism (ko00920)”, “Starch and sucrose metabolism(ko00500)” (p-value ≤ 0.05).

### 3.3 RNA-Seq analysis of passion fruit under Mg deficiency stress

**Figure 3:**
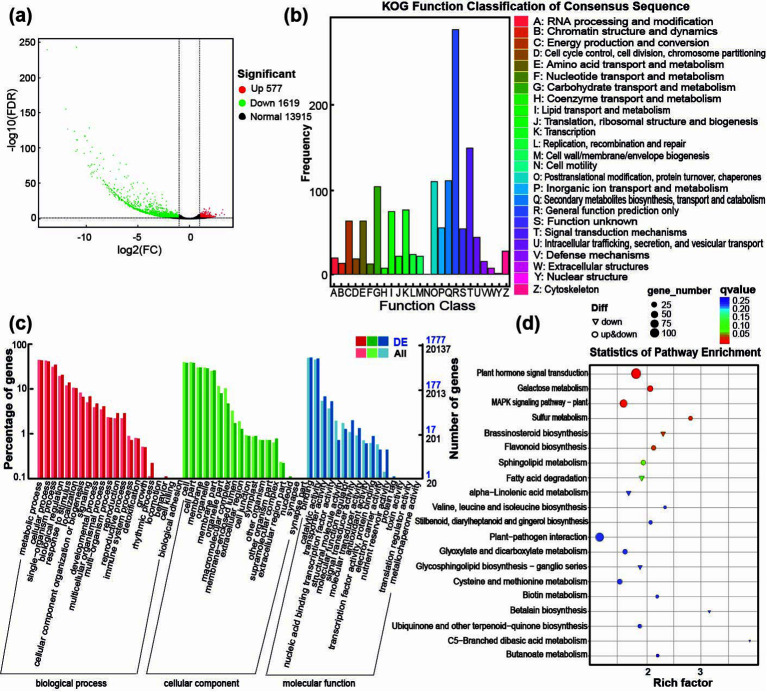
Analysis of differentially expressed genes (DEGs) in passion fruit under Mg deficiency. **(a)** Volcano plot of DEGs (Red dots indicate the upregulated genes, green dots indicating the downregulated genes, and black dots indicating the genes with no significant change.) **(b)** KOG functional classification **(c)** GO annotation **(d)** KEGG enrichment bubble chart (Inverted triangle indicates downward adjustment, and circle indicates both upward and downward adjustments. Color of graph represents the q-value. Size of graph reflects the number of distinct genes.)

According to differential expression analysis between sample groups, the genes with FDR < 0.05 and FC ≥ 2.0 were considered differentially expressed genes (DEGs) (**Supplementary Figure 2**). Under Mg deficiency stress, 2,196 DEGs were identified, with 577 genes upregulated and 1,619 genes downregulated (**Figure 3a**). The KOG functional classification analysis indicated that the unigenes could be categorized into 25 distinct groups. Notably, Group R, which pertains to general function prediction, was the most prominently represented category. Group T, associated with signal transduction mechanisms, also comprised a significant proportion of the genes, implying that magnesium deficiency may trigger intracellular signaling pathways that are potentially involved in magnesium sensing and homeostasis. Groups O, related to post-translational modification, protein turnover, and chaperones, and Q, concerning secondary metabolite biosynthesis, transport, and catabolism, ranked third in terms of gene proportion. Specifically, Group Q’s involvement in the biosynthesis of anthocyanins and flavonoids suggests that magnesium deficiency might influence fruit coloration and nutritional quality by modulating secondary metabolic pathways. Conversely, Groups Y, associated with nuclear structure, and N, related to cell motility, exhibited the lowest percentage of genes (**Figure 3b**). Gene ontology annotation analysis showed that 1,777 DEGs were distributed in 47 terms consisting of 18 biological processes, 16 cellular components, and 13 molecular functions. Within the biological process category, the functions related to reproduction and reproductive processes demonstrated significant proportional differences, suggesting that magnesium deficiency may adversely affect the reproductive development of passion fruit. In the cellular components category, terms such as organelle part, macromolecular complex, membrane-enclosed lumen, and extracellular region part exhibited notable proportional differences, which aligns with the magnesium deficiency-induced chloroplast structural damage (**Figure 1f**). In the molecular function category, structural molecule activity, signal transducer activity, and nutrient reservoir activity showed significant proportional differences, with genes associated with signal transducer activity potentially involved in the perception of magnesium deficiency and the activation of downstream adaptive responses (**Figure 3c**). These findings suggest that the enrichment pattern of DEGs diverges from that of all genes, warranting further investigation into these functions. To analyze the functions of DEGs under the stress of Mg deficiency, KEGG enrichment analysis was performed on DEGs between Mg deficiency and normal treatment (**Figure 3d**). The analysis revealed that DEGs were predominantly enriched in pathways such as plant hormone signal transduction (ko04075), galactose metabolism (ko00052), the MAPK signaling pathway specific to plants (ko04016), sulfur metabolism (ko00920), brassinosteroid biosynthesis (ko00905), and flavonoid biosynthesis (ko00941). Notably, galactose metabolism emerged as one of the most significantly enriched pathways, with key genes such as GOLS, RAFS, RPV1, and PGI1 exhibiting differential expression. These genes regulate the synthesis and accumulation of raffinose, melibiose, fructose, and succinate, which are crucial for osmotic adjustment and energy provision, thereby enhancing the adaptability of passion fruit to magnesium deficiency. Furthermore, the enrichment of the flavonoid biosynthesis pathway aligns with the observed reduction in anthocyanin content in the fruit peel under magnesium-deficient conditions (**Figures 2b–d**), implying that magnesium deficiency may downregulate flavonoid-related gene expression, consequently impacting fruit coloration. Although pathways directly associated with photosynthesis and chlorophyll metabolism were not detailed in this excerpt, their potential involvement remains an area for further exploration.

### 3.4 Combined analysis of LC–MS and RNA-Seq profiles under Mg deficiency

**Figure 4:**
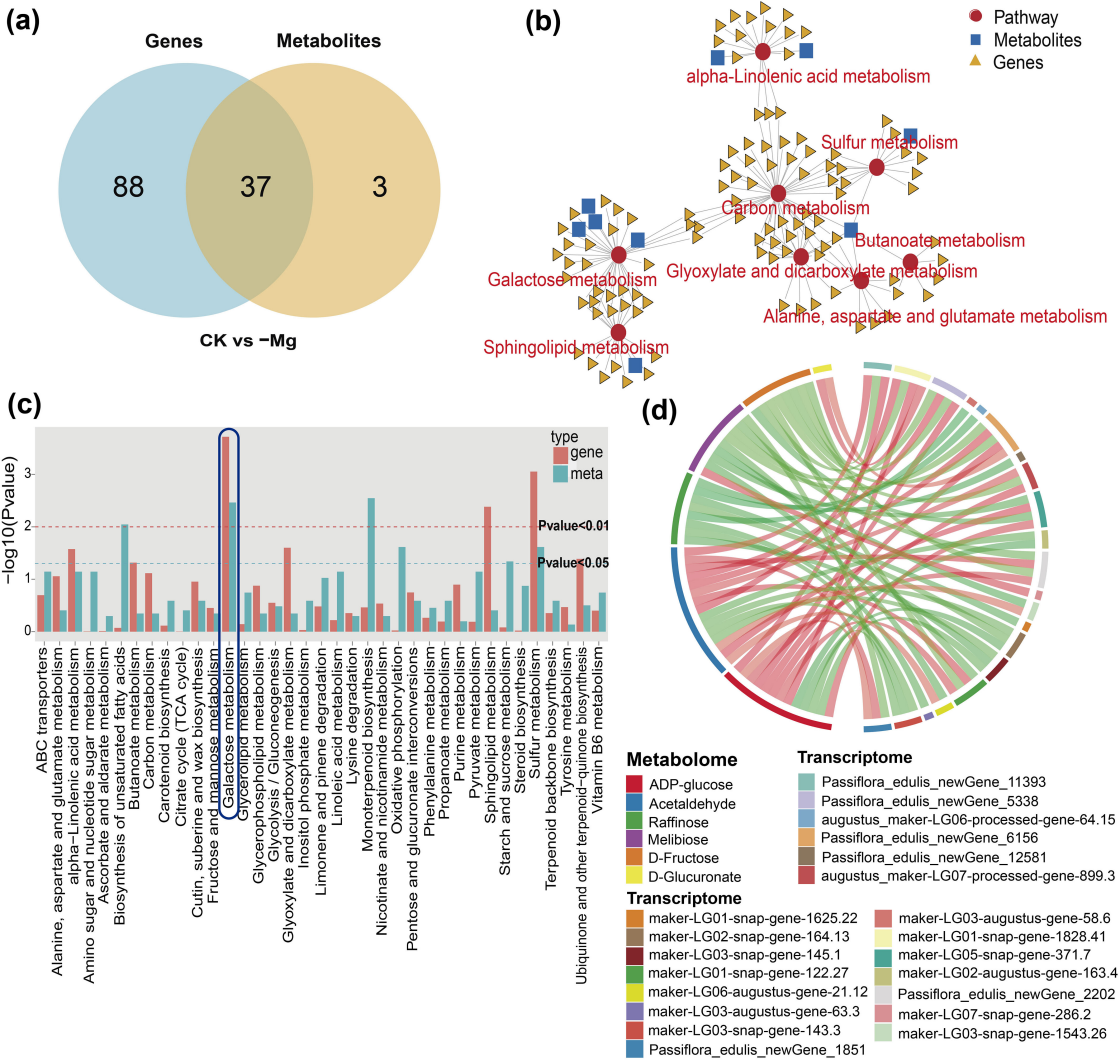
Network analysis of DEGs and DAMs in passion fruit under Mg deficiency. **(a)** Venn diagram of DEGs and DAMs (Blue circles indicating the number of genes, yellow circles indicating the number of metabolites.) **(b)** Network of DEGs and DAMs and pathways (Red dots indicating pathways, blue squares indicating metabolites, yellow triangles indicating genes.) **(c)** KEGG enrichment of DEGs and DAMs (P-value was calculated by a hypergeometric test, which indicated the degree of enrichment of DEGs or DAMs.) **(d)** Chord diagram of correlation network for galactose biosynthesis (Metabolites are shown on the left side of the circle, and genes are shown on the right side of the circle.)

To further clarify the relationship between genes and metabolites of passion fruit under Mg deficiency stress, the co-expression networks of transcriptome and metabolome were analyzed. Venn Diagram analysis showed that 37 KEGG pathways were enriched by both genes and metabolites (**Figure 4a**). In this study, eight metabolic pathways were found to be enriched with differential genes and metabolites: Galactose metabolism (ko00052), Carbon metabolism (ko01200), Alanine, aspartate and glutamate metabolism (ko00250), Butanoate metabolism (ko00650), Glyoxylate and dicarboxylate metabolism (ko00630), Sulfur metabolism (ko00920), alpha-Linolenic metabolism (ko00592), Sphingolipid metabolism (ko00600) (**Figure 4b**). The results showed that the KEGG enrichment analysis of differentially accumulated metabolites (DAMs) and DEGs were significantly enriched in the pathways of Galactose and Sulfur metabolites (p-value < 0.05) (**Figure 4c**). Among these pathways, only galactose metabolism exhibited strong concordance between DEGs and DAMs and showed extremely significant enrichment (p < 0.05). To further clarify the role of galactose metabolism in passion fruit pulp under Mg deficiency stress, the differential genes and metabolites involved in the galactose metabolism pathway were subjected to correlation network analysis (**Figure 4d**).

### 3.5 Comprehensive metabolic network analysis under Mg deficiency

**Figure 5:**
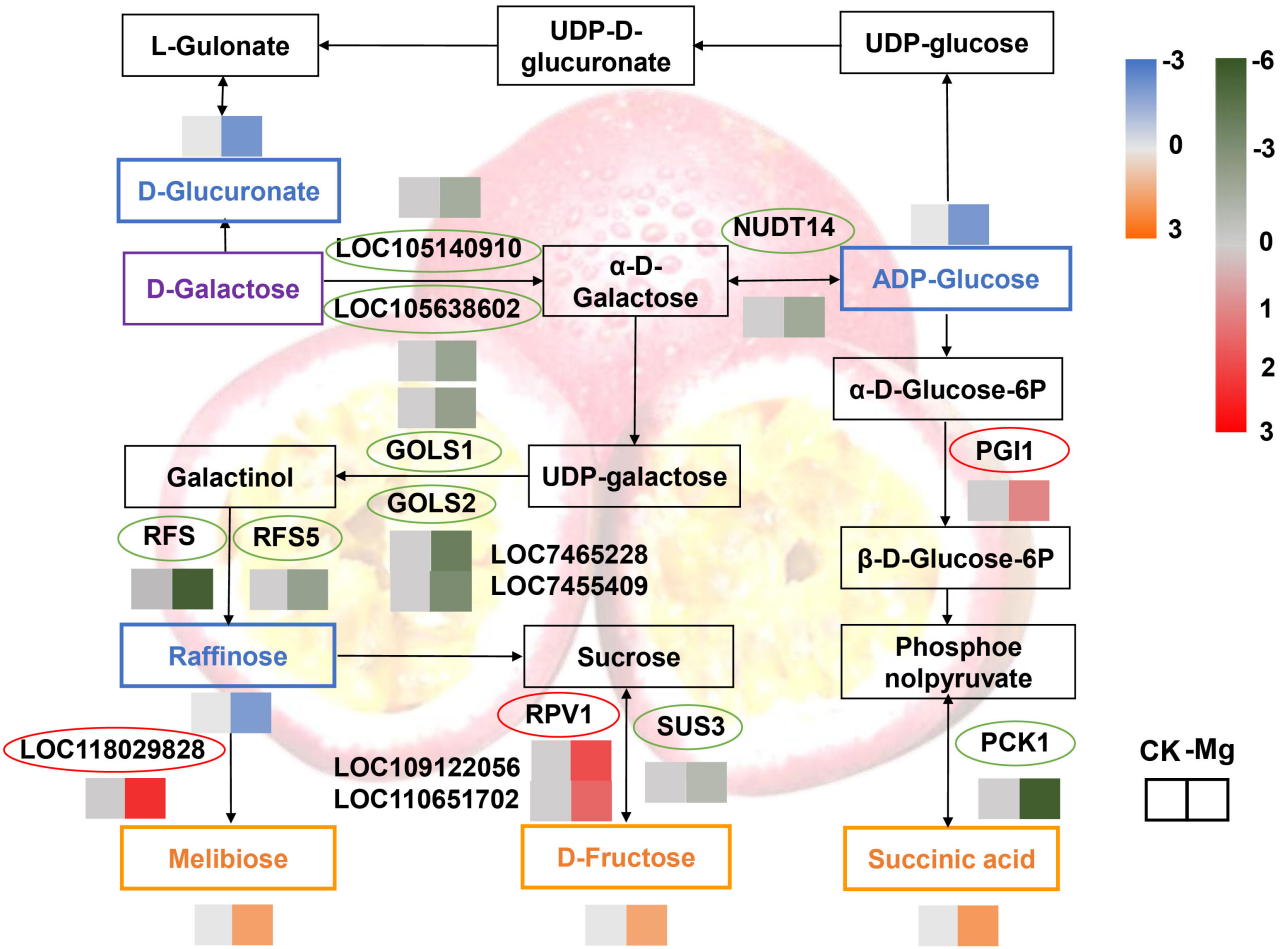
Analysis of galactose metabolic networks in the pulp of passion fruit under Mg deficiency stress. Heat maps depict changes in gene expression and metabolite abundance, with the Mg deficiency treatment (-Mg) on the left and the control treatment (CK) on the right. Metabolites labeled in black were not detected in this study. Rectangle shapes represent metabolites, with yellow indicating up-regulated metabolites, blue indicating down-regulated metabolites, and black indicating undetected in this study. Oval shapes represent genes, with green indicating up-regulated genes, and red indicating up-regulated genes.

Galactose metabolic pathway of passion fruit under Mg deficiency stress was constructed based on the KEGG pathway and published literatures, and differentially expressed gene and metabolites were integrated into the pathway-based map (**Figure 5**). Galactose is a simple carbohydrate that typically originates from glucose and can also bind with glucose, lipid, and proteins to form lactose, glycolipids, phospholipids, and glycoproteins (Shahbazy et al., 2020). In this study, 7 identified metabolites involved in galactose metabolism showed significant changes under normal Mg concentration, with 3 metabolites increased and 4 metabolites decreased compared with those under normal conditions. D-Glucuronate derived from D-Galactose decreased. The degradation of D-Galactose into α-D-Galactose was affected by the downregulation of *LOC105140910* (aldose 1-epimerase-like), and *LOC105638602* (aldose 1-epimerase). The α-D-Galactose was subsequently converted into UDP-galactose. UDP-galactose participates in the synthesis of galactinol together with myo-inositol; however, the downregulation of galactinol synthase genes (GOLS1 and GOLS2) reduced galactinol production. Consequently, raffinose synthesis was decreased due to the downregulation of raffinose synthase (RAFS contain RFS and RFS5). Moreover, raffinose was further metabolized into melibiose by the *LOC118029828* (UPF0481 protein At3g47200-like) gene. Under Mg deficient conditions, the conversion of sucrose to fructose may be associated with the enhancement of the RPV1 resistance gene. Another branch of the Leloir pathway uses α-D-glucose-1P as an important intermediate. Under Mg deficiency, the conversion of α-D-glucose-1-phosphate into ADP-glucose was reduced, accompanied by the downregulation of the nudix hydrolase 14 gene (NUDT14). In the last step of glycolysis, Phosphoenolpyruvate (PEP) is converted into pyruvate catalyzed by pyruvate kinase, a process that requires the participation of potassium and Mg ions. Due to Mg deficiency, pyruvate production was significantly reduced, resulting in decreased formation of the fruit-aroma compound acetaldehyde. Consequently, the metabolic flux was reoriented towards glycogenesis to facilitate the production of oxaloacetate. The upregulated expression of PGI1, a critical gene involved in the synthesis and accumulation of soluble sugars, may indirectly enhance the synthesis and accumulation of succinic acid. This is achieved through the increased production of β-D-glucose-6-phosphate, an intermediate in the succinic acid biosynthetic pathway. In summary, Mg deficiency accelerates raffinose hydrolysis and promotes glycogenesis within the galactose metabolic pathway.

## 4 Discussion

### 4.1 Mg deficiency disrupts leaf structure and inhibits photosynthesis, impairing product export in leaf of passion fruit

Mg is an essential element that serves as the central atom of the chlorophyll molecule within the porphyrin ring and is crucial for the structure of thylakoid membranes and the stacking of grana (Zhou et al., 2011; Dos Santos et al., 2023). Magnesium deficiency disrupts the cellular ultrastructure of plant leaves (such as chloroplasts, cell membranes, and cell walls), causing reduced grana stacking and accumulation of plastoglobuli, as observed in many plant species (Shi et al., 2024; Zhu et al., 2025) as well as in the leaves of passion fruit in this study (**Figure 1f**) (Shi et al., 2024).Such structural degradation directly impairs photosynthetic carbon assimilation. Damage to chloroplast structure directly affects plant photosynthesis (Ye et al., 2019). Magnesium-deficient leaves exhibit significant decreases in SPAD value, net photosynthetic rate, and transpiration rate, along with impaired chlorophyll synthesis and a reduced chlorophyll-to-xanthophyll ratio, ultimately leading to leaf yellowing. In *Vicia faba* L. leaves subjected to Mg deficiency, a decline in SPAD values, net photosynthetic rate, and transpiration rate was observed, accompanied by an increase in sucrose content and starch accumulation (Neuhaus et al., 2013; Parisa et al., 2025). Similarly, the magnesium-deficient passion fruit leaves in this study also exhibited significant decreases in SPAD value and net photosynthetic rate, along with leaf yellowing (**Figures 1b, c**). The disruption of photosynthesis further limits the translocation of photosynthetic products, such as sucrose and starch, resulting in their excessive accumulation within the leaves. This phenomenon has been documented across various plant species, including mulberry, tomato, citrus, faba bean, and tobacco (Neuhaus et al., 2013; Yang et al., 2019; Ullah et al., 2022; Wang et al., 2022). For instance, in tobacco, Mg deficiency resulted in significant starch granule accumulation and elevated sucrose and starch content, particularly noticeable after seven days of treatment (Chen et al., 2025a). Similarly, magnesium-deficient passion fruit leaves have demonstrated elevated levels of starch and soluble sugars (**Figure 1d**). In conclusion, magnesium deficiency adversely affects chloroplast structure, impairs photosynthesis, and triggers metabolic disorders, culminating in reduced carbon assimilation and the abnormal accumulation of photosynthetic products.

### 4.2 Mg deficiency reduces the anthocyanin/Chlorophyll ratio in peel and the key aroma-active compounds in pulp

Mg availability plays a crucial role in modulating fruit coloration. In citrus, a 180-day Mg treatment consistently reduced chlorophyll a, chlorophyll b, and total chlorophyll content. The treatment with Mg application led to reduced chlorophyll levels in the flavedo of Satsuma mandarin (165–195 days after flowering) and navel orange (150–159 days after flowering), which was achieved by down-regulating key chlorophyll synthesis genes (GGDR, CHLH, CHLM, CHL27, PORA) while simultaneously up-regulating major chlorophyll degradation genes (NYC, PPH, PAO, RCCR) (Liu et al., 2025c). The application of Mg in apple orchards was found to significantly increase anthocyanin concentration in the fruit peel and upregulate the expression of key biosynthesis genes (MdCHS, MdF3H, MdMYB1, MdbZIP44) (Tian et al., 2023). Additionally, a separate study on olive trees demonstrated a biphasic response in leaf chlorophyll content to the duration of Mg treatment, initially increasing before declining with extended exposure (Saidana et al., 2009). In the present study, Mg deficiency was found to significantly decrease anthocyanin content while increasing chlorophyll content in passion fruit peel, resulting in a reduced anthocyanin-to-chlorophyll ratio (**Figures 2b–d**). These results are consistent with previous reports in other fruit species, such as citrus and apple, where Mg availability modulates peel coloration by affecting the synthesis and accumulation of anthocyanins and chlorophyll (Liu et al., 2022, 2023a; Tian et al., 2023; Chen et al., 2025b). Our findings extend this understanding to passion fruit, highlighting a conserved role of Mg in regulating fruit peel pigmentation.

Mg is integral to the development of fruit aroma. Prior research has demonstrated that plant growth regulators can augment Mg uptake in grapes, subsequently affecting their aromatic profile (Wang et al., 2025b). In rice, the foliar application of Mg fertilizer has been shown to significantly enhance the biosynthesis of the key aroma compound 2-acetyl-1-pyrroline (Hassan et al., 2024). Additionally, numerous studies suggest that Mg effectively intensifies the sweet, floral, and fruity notes in tea, thereby enhancing its overall fragrance (Liu et al., 2025a, b; Ye et al., 2025). Notably, research conducted by Jianghua Ye et al. revealed that Mg treatment markedly increases the geraniol content in tea leaves (Ye et al., 2025). These findings are consistent with the results of the current study, which observed that Mg deficiency resulted in a reduction in the relative content of several persistent aroma metabolites, specifically geraniol and (2E,6E)-farnesol (**Figure 2h**). These compounds contribute rose and lily-of-the-valley floral notes, respectively, suggesting a potential decrease in sweet and floral aromas suggesting a potential. It should be noted that the conclusions regarding aroma changes in this study are based solely on the analysis of relative contents of compounds such as geraniol and farnesol. Although these compounds have been recognized as major contributors to floral and sweet notes, their reduced levels do not necessarily imply a significant attenuation of fruit aroma. Future studies should incorporate sensory evaluation panels or olfactometric analyses to verify the actual sensory impact of magnesium deficiency on passion fruit aroma.

### 4.3 Regulation of galactose metabolism enhances Mg deficiency stress resistance in passion fruit via biosynthesis of raffinose, fructose and succinate

Galactose metabolism has been associated with plant resistance to various abiotic stresses, including low temperature (Shu et al., 2025), drought (Shahbazy et al., 2020; Singh et al., 2023; Kudapa et al., 2024; Ge et al., 2025), and saline-alkali (Wang et al., 2021) conditions. Prior research has shown that low-temperature stress significantly induces the galactose metabolism pathway in plants, such as in the leaves of Tunisian soft-seed pomegranate (Punica granatum L.) (Guan et al., 2023) and seedlings of Xanthoceras sorbifolia (Wang et al., 2020). Additionally, in peaches, cold stress activates this pathway, leading to a significant accumulation of metabolites such as galactose, raffinose, glucose, and fructose in cold-tolerant cultivars compared to cold-sensitive ones (Li et al., 2023). In chickpea (*Cicer arietinum* L.), drought stress primarily activates the expression of proteins involved in antibiotic biosynthesis, galactose metabolism, and isoflavonoid biosynthesis, with six metabolites—fructose, galactose, glucose, galactinol, myoinositol, and raffinose—being significantly associated with galactose metabolism (Kudapa et al., 2024). The response of foxtail millet (*Setaria italica* L.) to saline-alkali stress is regulated through various pathways, including MAPK signaling, glycerolipid metabolism, phosphatidyl and phosphate metabolism, galactose metabolism, and endoplasmic reticulum protein processing (Wang et al., 2025a). This study suggests that under Mg deficiency, passion fruit may employ the galactose metabolism pathway as a means of adaptation, thereby representing a potential adaptive mechanism (**Figure 4**).

Galactinol synthase (GOLS) serves as the pivotal enzyme catalyzing the initial committed step in the biosynthesis of raffinose (Ren et al., 2022; Liu et al., 2023). Prior research has established that exposure to cold conditions stimulates the expression of the GOLS and raffinose synthase (RAFS) genes in alfalfa, thereby modulating the galactose metabolic pathway (Zhang et al., 2024). Under conditions of low-temperature stress, the expression of GOLS is significantly up-regulated in cold-tolerant peach varieties, while it remains largely unchanged or only slightly induced in cold-sensitive varieties (Li et al., 2023). These findings collectively highlight GOLS and RAFS as critical genes in the activation of galactose metabolism under low-temperature stress. Furthermore, the study by Tang et al. demonstrates that the coordinated high expression of sucrose synthase (SUS) and phosphoglucoisomerase (PGI) in passion fruit enhances the metabolic flux from sucrose to fructose and among hexose phosphates, thereby facilitating the synthesis and accumulation of soluble sugars (Tang et al., 2025). In this study, it was observed that Mg deficiency leads to the downregulation of GOLS expression. This finding indicates that passion fruit may influence galactose metabolism by inhibiting the expression of both GOLS and raffinose synthase (RAFS), consequently decreasing raffinose biosynthesis. Under Mg deficiency, the expression of *LOC118029828* and RPV1 (*LOC109122056* and *LOC110651702*) was found to be upregulated, while the expression of SUS3 was downregulated, thus facilitating the conversion of raffinose into melibiose and D-fructose. Furthermore, Magnesium deficiency stress enhances the expression of PGI1, a critical gene implicated in the synthesis and accumulation of soluble sugars. PGI1 facilitates the production of β-D-glucose-6-phosphate, an intermediate in succinate biosynthesis. Consequently, the upregulation of PGI1 expression may indirectly facilitate the synthesis and accumulation of succinate. In passion fruit, D-fructose serves as a vital energy source for growth and stress responses, while melibiose and succinate function as critical regulators in response to Mg deficiency stress, with their concentrations being highly responsive to abiotic stimuli (Agrawal et al., 2013; Song et al., 2018; Shi et al., 2022). Under conditions of phosphorus deficiency, rice demonstrated a notable enhancement of galactose metabolism in its leaves, accompanied by a significant accumulation of sucrose, trehalose, and melibiose (Yan et al., 2022). Meanwhile, green soybean exhibits adaptive mechanisms to low-temperature stress by modulating pathways associated with galactose metabolism as well as ascorbate and aladarate metabolism, wherein sucrose, lactose, and melibiose are likely contributors to its cold tolerance (Lin et al., 2024). A comparative analysis revealed that the waterlogging-tolerant cotton cultivar exhibited elevated levels of sinapyl alcohol, L-glutamic acid, galactaric acid, glucose 1-phosphate, L-valine, L-asparagine, and melibiose relative to the sensitive cultivar (Lin et al., 2024). Under drought stress conditions, Salvia miltiorrhiza leaves demonstrated increased accumulation of succinic acid and D-glucose (Zhang et al., 2021). Consequently, these findings indicate that under Mg deficiency, passion fruit upregulates the expression of *LOC118029828* and *PGI1*, which may indirectly contribute to the synthesis and accumulation of melibiose and succinic acid in the pulp, potentially serving to alleviate Mg stress (**Figure 5**).

**Figure 6:**
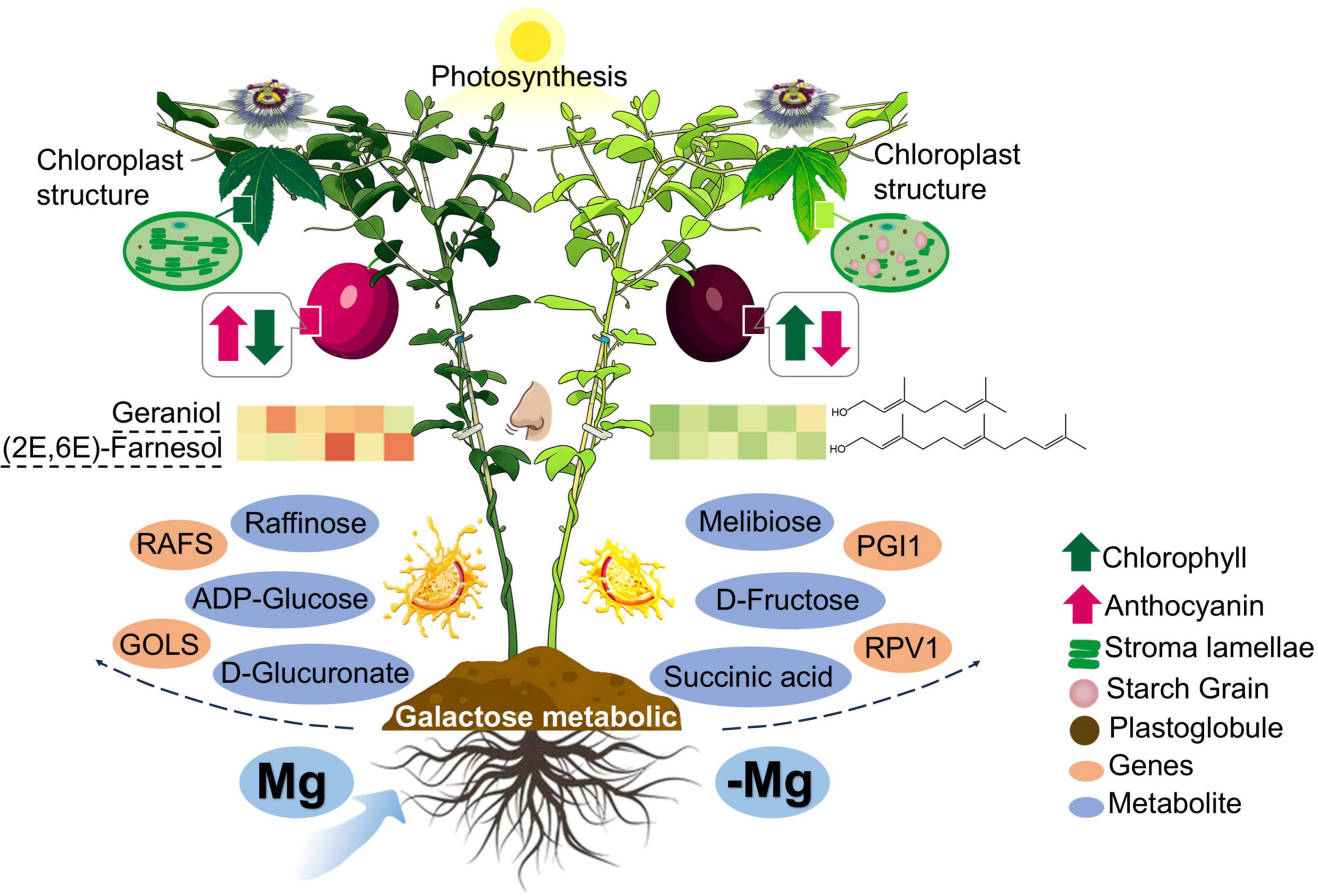
The conceptual framework of physiological responses and mechanisms in passion fruit under Mg deficiency. Elements depicted with solid lines are substantiated by quantitative data, including physiological measurements, ultrastructural analysis, and pigment content assessments. Elements represented with dashed lines, including genes, metabolites, and galactose metabolic pathways, indicate relative-level changes as observed in transcriptomic and metabolomic studies, as well as potential pathways.

In conclusion, this study employed a comprehensive approach that integrated analytical chemistry, metabolomic, and transcriptomic analyses to systematically elucidate the complex effects of Mg deficiency on passion fruit. This investigation encompassed various aspects, including leaf physiology, peel coloration, pulp aroma, and the fruit’s underlying mechanisms in response to Mg deficiency stress. Furthermore, by comparing the findings with existing literature, a theoretical framework (**Figure 6**) was proposed to illustrate the coordinated physiological and metabolic responses that occur in passion fruit under Mg deficiency conditions. Nonetheless, this theoretical framework necessitates additional validation. Subsequent research should prioritize the quantitative analysis of key metabolites and enzymatic activities, alongside *in vivo* functional validation of genes involved in pertinent pathways. This can be achieved through methodologies such as gene editing, overexpression, or mutant analysis, to more accurately elucidate the molecular regulatory network under magnesium deficiency stress. Moreover, the integration of multi-omics data with quantitative models derived from biochemical assays will augment the predictive accuracy and generalizability of this framework.

## 5 Conclusions

This study investigated the physiological and molecular mechanisms by which Mg deficiency impairs growth and fruit quality in passion fruit. The results indicate that Mg deficiency disrupts chloroplast structure, inhibits photosynthesis, and leads to abnormal carbohydrate accumulation in leaves. Simultaneously, it reduces the anthocyanin-to-chlorophyll ratio in the fruit peel, thereby delaying color and diminishing marketability. Notably, Mg deficiency specifically correlates with changes in the galactose metabolism pathway, accompanied by elevated biosynthesis of critical metabolites such as melibiose and succinate, which seem to play roles in osmotic adjustment and energy provision under stress conditions. These findings contribute to a deeper understanding of Mg’s role in regulating photosynthesis and stress metabolism in fruit crops and offer potential markers for diagnosing Mg deficiency. In conclusion, this research elucidates key metabolic responses to Mg deficiency in passion fruit, providing a scientific foundation for improving Mg nutrition management and fruit quality in sustainable agriculture.

## Data Availability

The datasets presented in this study can be found in online repositories. The names of the repository/repositories and accession number(s) can be found below: https://ngdc.cncb.ac.cn/gsa, GSA: CRA030244.
