# Supplementary material for: Multi-omics analysis reveals galactose metabolism as a key regulatory pathway of stress adaptation to magnesium deficiency in passion fruit
Source: Front Plant Sci. 2026 Jun 26;17:1878176. doi: 10.3389/fpls.2026.1878176 (PMC13349930; doi:10.3389/fpls.2026.1878176)
Supplement: Supplementary file 1 [file SupplementaryFile1.doc]

***Supplementary Material***


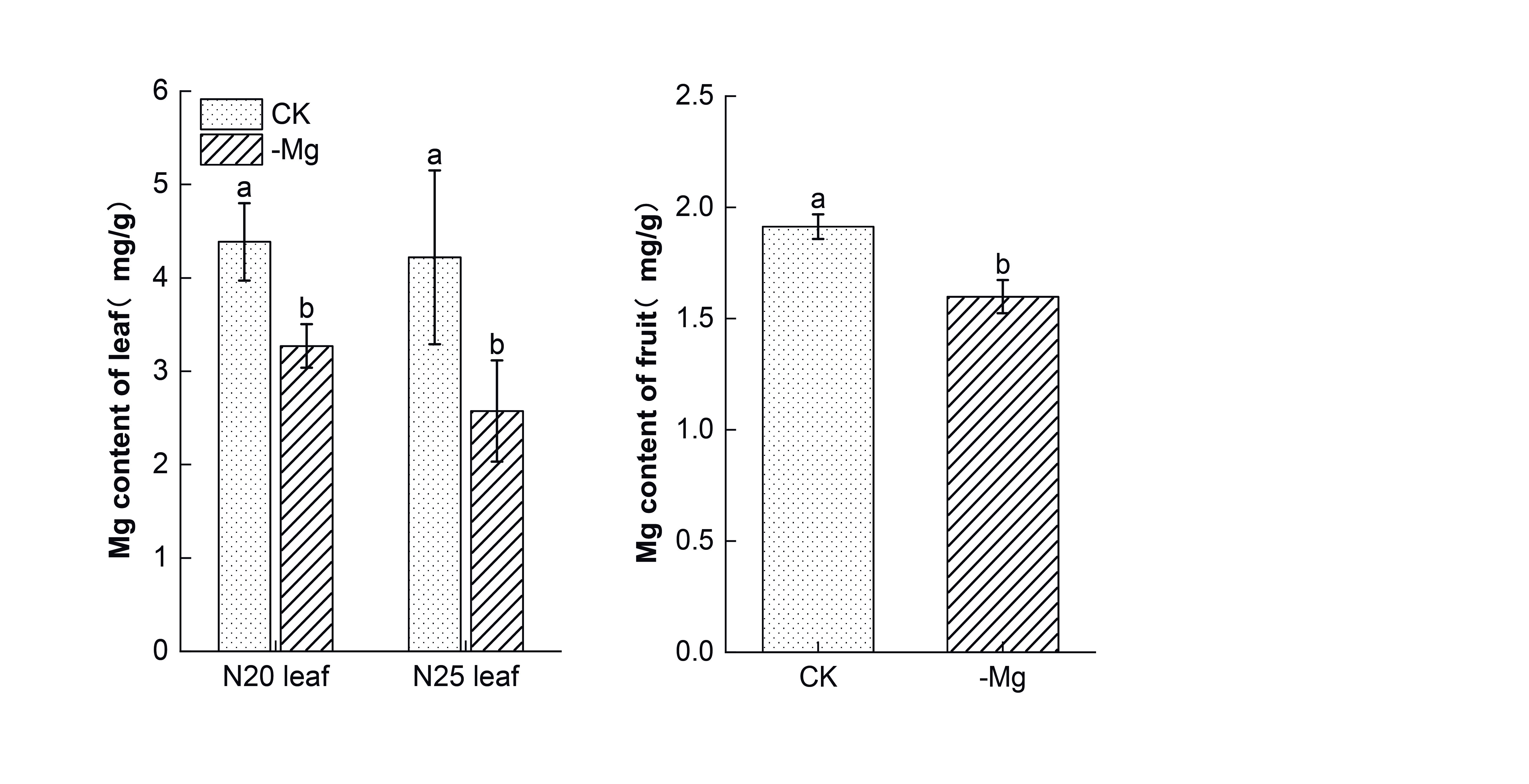


**Supplementary Figure 1.** Mg content of passion fruit in leaves and fruit under different concentrations of magnesium treatments. (a) Mg content of the 20th and 25th leaf (b) Mg content of passion fruit


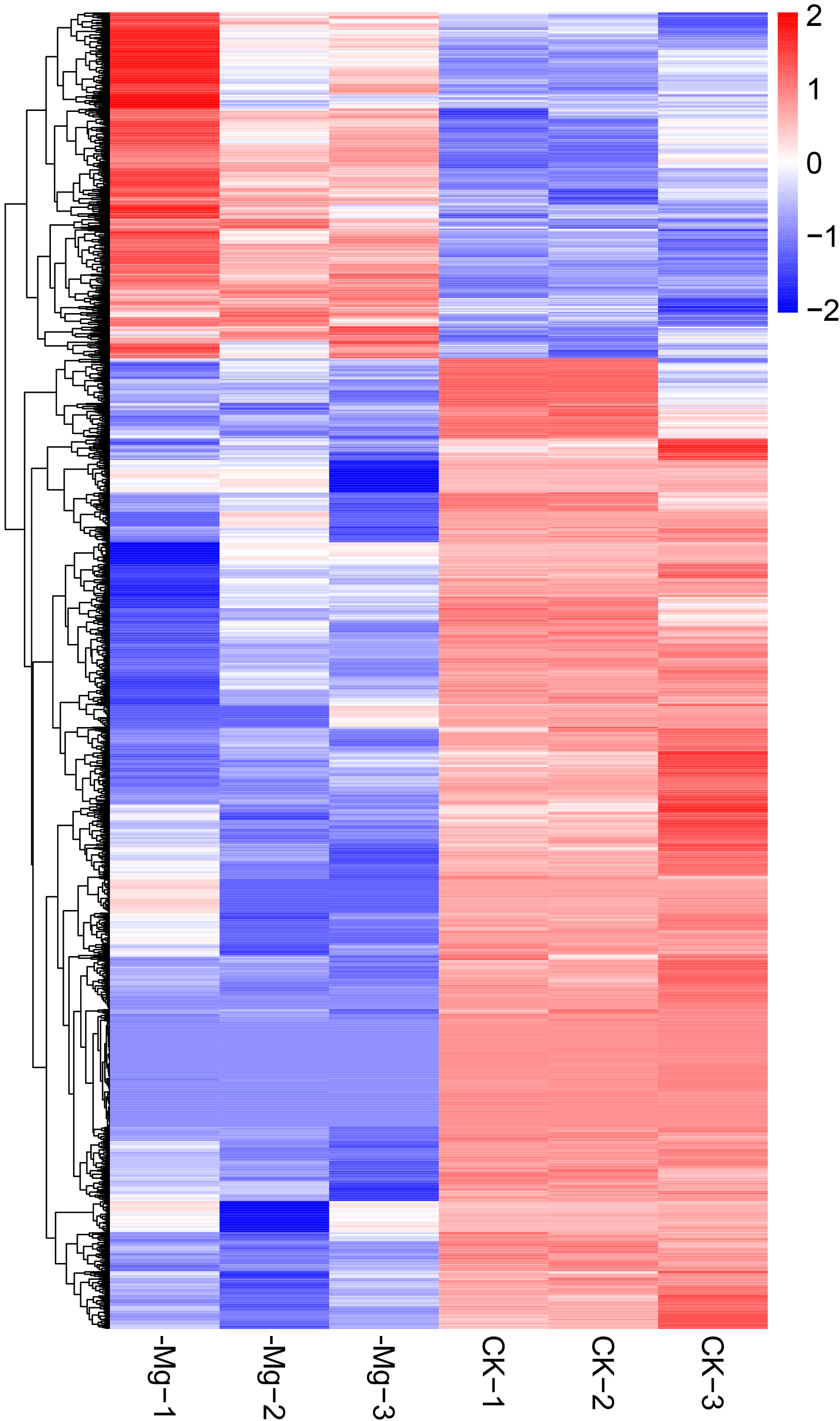


**Supplementary Figure 2.** Heatmap of expression of genes associated with different concentrations of magnesium treatments.


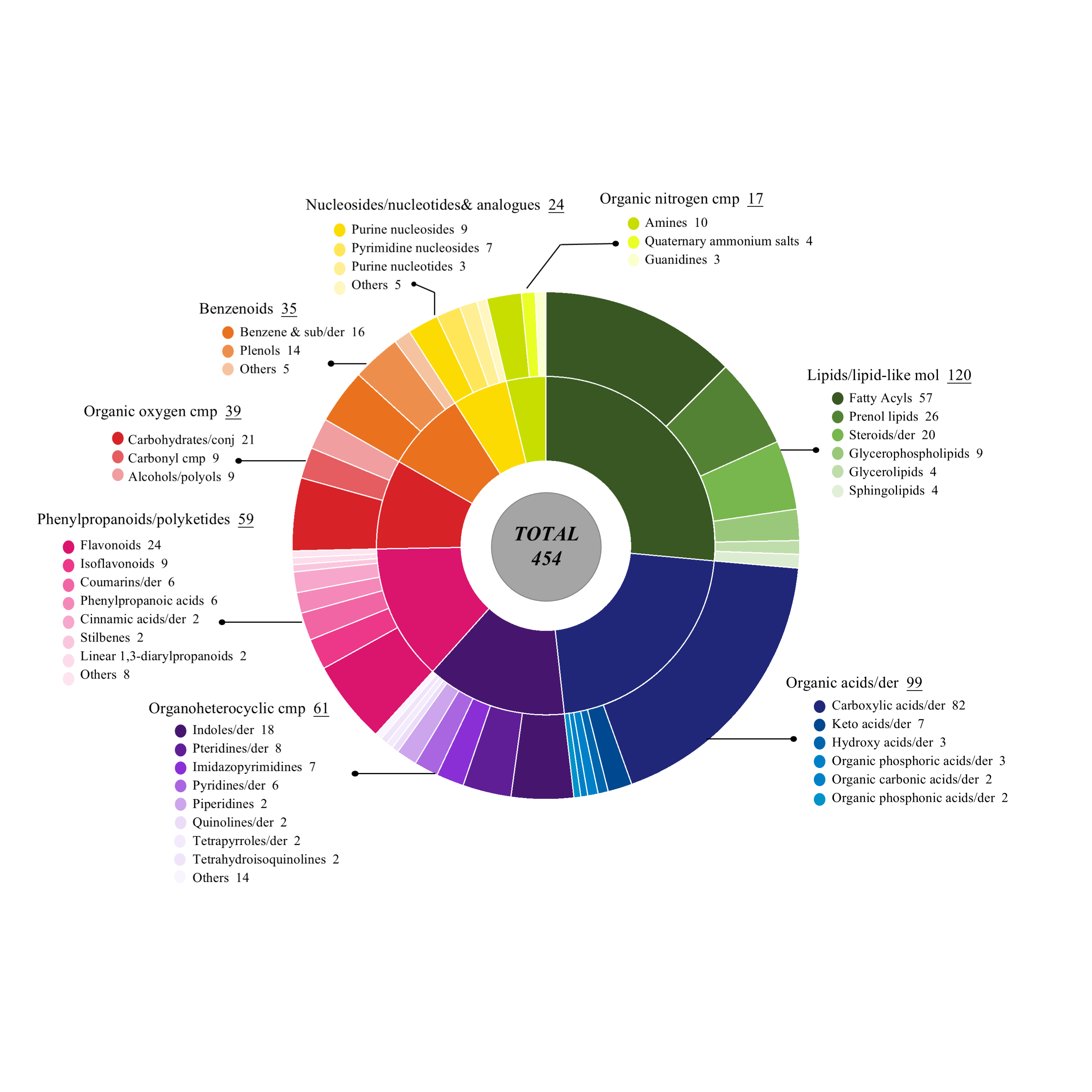


**Supplementary Figure 3.** Compositional proportions of metabolite classes identified in passion fruit pulp.
